# Epithelioma of Malherbe: new ultrasound patterns

**DOI:** 10.1186/1756-9966-29-42

**Published:** 2010-05-06

**Authors:** Francesco M Solivetti, Fulvia Elia, Alessandra Drusco, Chiara Panetta, Ada Amantea, Aldo Di Carlo

**Affiliations:** 1 Struttura di Radiologia e Diagnostica per Immagini, Istituto Dermosifilopatico di Santa Maria e San Gallicano-IFO-Roma, Italy; 2 Struttura di Dermopatologia, Istituto Dermosifilopatico di Santa Maria e San Gallicano-IFO-Roma, Italy; 3 Direttore Scientifico, Istituto Dermosifilopatico di Santa Maria e San Gallicano -IFO-Roma, Italy

## Abstract

**Backround:**

Calcifying epithelioma of Malherbe, or Pilomatricoma, is considered an uncommon cutaneous neoplasia, normally occurring in children as a solitary, firm, asymptomatic, hard, subcutaneous, slowly growing nodule on the face, neck, or proximal upper extremity. In literature, two Pilomatricoma ultrasound patterns are described: the totally calcified nodule and the hypoechoic nodule with internal calcific foci. High frequency ultrasound has not yet been applied for routine diagnosis of Pilomatricoma. The aim of the study was to retrospectively identify specific ultrasound features.

**Methods:**

We retrieved 124 histologically Pilomatricoma cases: 28 patients with 32 lesions were preoperatively evaluated with ultrasound.

**Results:**

22/32 have shown a solid formation, hypoechoic, with a sharp outline. Of these 22, 10 lesions were completely calcifying and 12 partially calcified. In 3/32 lesions with uncertain diagnosis, ultrasounds showed a complex/mixed pattern with pseudo-fluid areas and microspots. 7/32 lesions with US different diagnosis included 3 complex lesions, 2 cystic lesions and 2 solid nodular lesions.

**Conclusion:**

In addition to well-known ultrasound patterns (completely calcified and partially calcified) we identified three new, not yet described, patterns that constitute the 31% of the cases: complex, pseudocistyc and pseudotumoral.

## Background

Calcifying Epithelioma of Malherbe - or Trichomatricoma, Pilomatricoma, Pilomatrixoma (PM) - is an uncommon tumour [1], with an incidence of 1/800-1000 cutaneous tumours and about 20 new reports per year [2,3], affecting predominantly women. It is more common at a young age, especially in the first two decades of life, with an onset below 10 years in 40% of cases [4,5]. Although multiple localizations have been described in literature [6,7], PM occurs as a solitary lesion on the face (47% of cases), neck [8] and upper trunk and can be associated to other diseases, e.g. Steinert's Myotonic Dystrophy and Gardner Syndrome [4,7,9,10].

Recent studies have shown that recurrent activating mutations in the ss-catenina gene (CTNNB1), induce PM tumourigenesis through activation of the WNT signalling pathway [11,12]. Despite the benign biological behaviour of the majority of cases, the treatment is still surgical. However, in recent years, aggressive cases with local post-surgery recurrences or metastasis have been described [2,3,13,14], accounting for variable percentage rates in literature, with 6 cases out of 228 in the Forbis series [6]. According to some authors [13], local recurrences are related to tumour aggressiveness, while for others, these cases are only associated with an incomplete surgical excision [15].

The tumour presents as a slow growing subcutaneous mass, sometimes dark on the surface, with well-defined borders and, often, with lobulated contours at ultrasound. The size of the tumour is usually small, less than one cm, but, in the Darwish series, 3 out 26 had more than 2 cm lesions and 11 out of 26 had 11 - 20 mm lesions [16].

Histologically, the lesion appears as a well defined nodule, often calcified and inflamed, sometimes reproducing a granulomatous reaction. It originates from the matrix cells of the hair follicle, having a basaloide appearance, composed of anucleated eosinophilic cells (shadow or ghost cells) which are typical of trichilemmal keratinization [17].

The clinical diagnosis is often difficult: in a recent series, most of the cases were clinically confused with sebaceous cysts [16] and, in the Pirouzmanesh series, only 100 out 346 (28,9%) cases were correctly diagnosed as PM [18].

In a survey where "soft rays" were employed, data did not discriminate among the different pathologies [19]. Although the finding of a nodular calcified lesion in children, should address the clinical diagnosis to PM [20], classic X-ray, ultrasound and CT scan do not allow a differential diagnosis with dermatofibroma, epidermoid cyst, lipoma, sebaceous calcified cyst, fibrocalcified lymphadenitis, foreign body granuloma, chronic abscess or organized hematoma [18,21].

Moreover, FNAB has shown a significant number of false positives and negatives [22] and MRI is considered inconclusive [23]: in the Lim series [20], out of 5 cases considered, only 60% were diagnosed correctly.

Therefore, it is necessary to identify a diagnostic imaging technology to assure a correct diagnostic hypothesis.

High-frequency ultrasound [24] is a very simple, reliable imaging technique, yet poorly reported in literature and in numerically limited series [19]. Hughes et al. [25] presented a cohort of 28 clinically suspected PM cases, diagnosed employing a relatively low frequency probe (7 MHz). 20 patients underwent surgery and were evaluated histologically: 16 were confirmed as PM, 2 were epidermoid cysts and, in 2, it was not possible to asses any diagnosis. Similar data have been reported by Ulrich et al. [26], Lim el al. [20], Hwang el al. [27] and Whittle el al. [28]; Buchwald et al. [29] diagnosed one case of PM using ultrasound microscopy. In the Whittle series [28], typical PM sonographic features were characterized by a hypoechoic small superficial nodule (between epidermis and dermis), with not always well-defined margins, with some calcified areas (98% of this series) of variable appearance, formed of central or peripheral single or grouped foci of variable shapes [24]. The lesion was sometimes surrounded by a hypoechoic halo and sometimes perilesional Doppler flow signals were present.

So far, two different PM sonographic patterns have been described in literature: the totally calcified nodule and the hypoechoic nodule with internal calcified foci.

Conducting a retrospective study of our cases, the paper aims to identify high-frequency ultrasound patterns of PM that should improve clinical diagnosis.

## Methods

Images of 124 patients with a histological diagnosis of PM were retrieved from the 1996-2008 archive of the Dermatopathology Unit of our Institute. Pre-operatory ultrasound images of 28/124 patients were available. In order to avoid the comparison of two inhomogeneous groups, we only analyzed data of these 28 patients (with 32 lesions and 5 different locations on one patient), whose clinical records were complete.

Fourteen females and 14 males, aged between 12 and 58 years, were considered in the study. Three different Esaote ultrasound units (Genoa, Italy) were sequentially used during the period 1996-2008: respectively, AU4 apparatus with 20-MHz Anular Array, single crystal probe, an AU5 apparatus, with the same probe, and, lastly, a My Lab 70, with linear probe having a maximum rated frequency of 18 MHz, completed of colour, power and pulsed Doppler.

Only in two cases, with unclear ultrasonographic features, was the study also performed with contrast medium (Sono Vue ^© ^Bracco, Milano, Italy), employing a multi-frequency linear probe (7-12 MHz); in all cases, retrospectively, the images were jointly discussed by Radiologists of our group.

Finally, data were analyzed using a statistical package IBM SPSS, limited by an obvious lack in the numbers of the cohort and the control group. Statistical analysis of data was performed by means of Mc Neman's test for binomial data to assess differences in sensitivity and specificity.

## Results

We reviewed 32 high-frequency ultrasound images of 28 patients (one patient had 5 lesions). Three different ultrasound units have been used sequentially during the period 1996-2008. The first two types of equipment, AU4 and AU5, which had the same probe, did not show any relevant image quality difference. Although using a slightly lower frequency with respect to the previous ones (18 MHz versus 20 MHz), the third apparatus, a My Lab70, showed a better image quality when the lesion size was compatible to the piezoelectric crystal resolution power.

The size of the 32 lesions ranged from 3 to 22 mm. In particular, 2 cases exceeded 20 mm, 6 were between 10 and 20 mm and the remaining 24 were smaller than 10 mm.

In 20 cases, the lesions were localized on the head, 2 on the neck, 8 on the forearm, in 1 case on the wrists and one on the back (Table 1 - Location of pilomatricoma).

**Table 1:** Locations of pilomatricomas

Localization	No. of lesions
*Head*	20
*Upper extremity*	8
*Neck*	2
*Wrist*	1
*Trunk*	1

We compared each clinical ultrasonographic diagnosis to the respective definitive histopathological response of the lesions. 22/32 cases (69%) were correctly diagnosed as PM, 7/32 cases (22%) were misdiagnosed and in 3/32 cases (9%), it was not possible to assess any diagnostic hypothesis with ultrasound.

**Figure 1:**
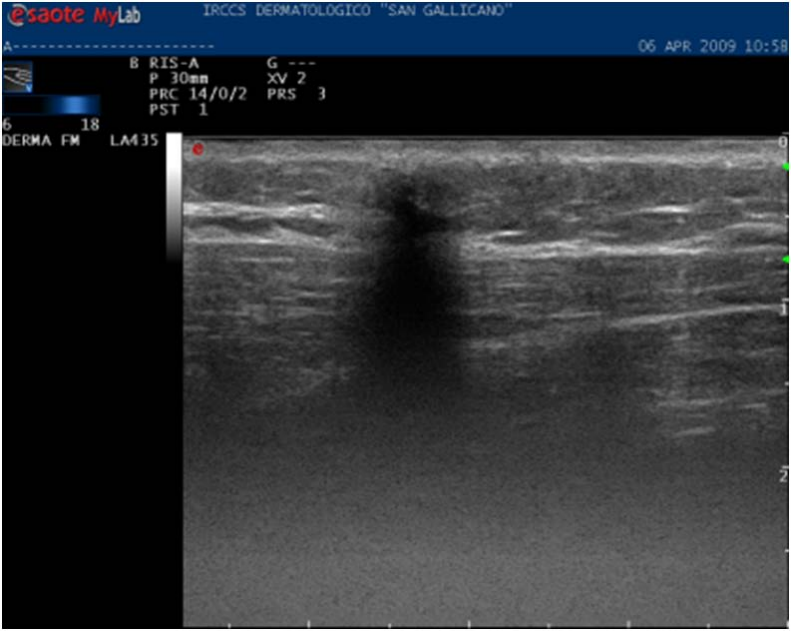
**Pattern type 1: nodulation fully calcified, no longer evaluable**.

**Figure 2:**
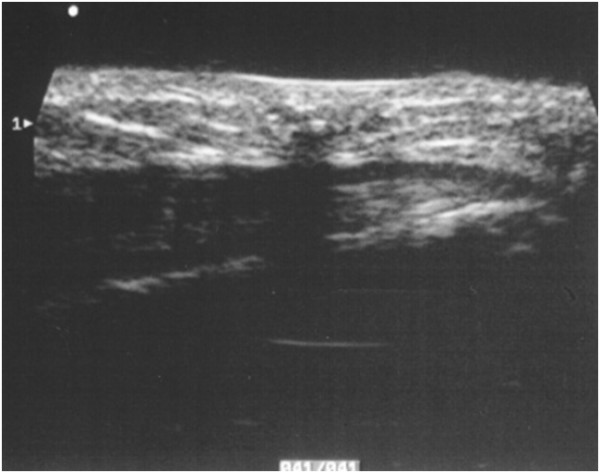
**Pattern type 2: partially calcified nodulation, mostly solid, hypoechogenic, with well defined borders, and coarse calcifications**.

In 4 cases, vascular signals were visible with colour and power Doppler; this feature was usually peripheral and only rarely intra-lesional, and was observed in lesions larger than 10 mm. The apparatus setting was that generally used for superficial lesions at low flow speed. Tumour locations were always superficial, between the dermis and subcutaneous tissue. Our ultrasound images, obtained with high-frequency probes, in all correctly diagnosed cases, showed solid, hypoechoic, and sharp rimmed lesions: 10 were fully calcified (Fig. 1) and 12 partially calcified (Fig. 2); 5 of the latter had only calcified microspots. In 4 cases, a perilesional peripheral hypoechoic halo was also observed.

**Figure 3:**
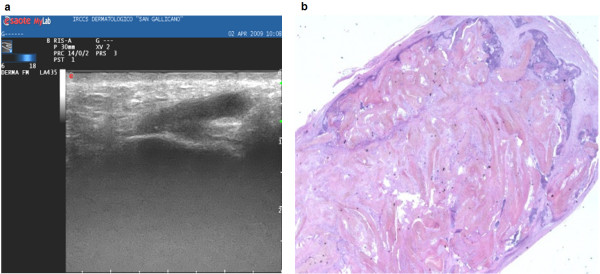
**Pattern type 3: complex nodulation, with undetectable contours, with fluid and macrocalcified areas**. The lesion presents well defined  borders. B) Histologic section at low power. The proliferation is surrounded  by connectival stroma, and is edged by a basaloid epithelia with  tricholemmal and shadow cells, associated to a moderate inflammatory  reaction (E-E1, 25x).

**Figure 4:**
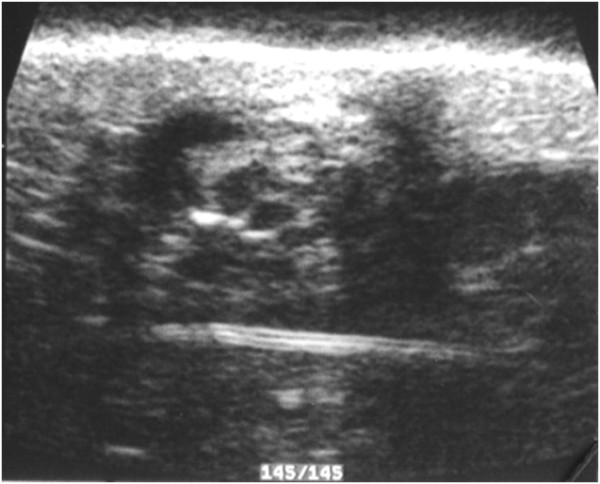
**Pattern type 4: A)Pseuso-cystic, Lesion borders and sizes are not well evaluable.** Fluid nodule with feature similar to a thickened wall  cyst, extending up to the derma.

**Figure 5:**
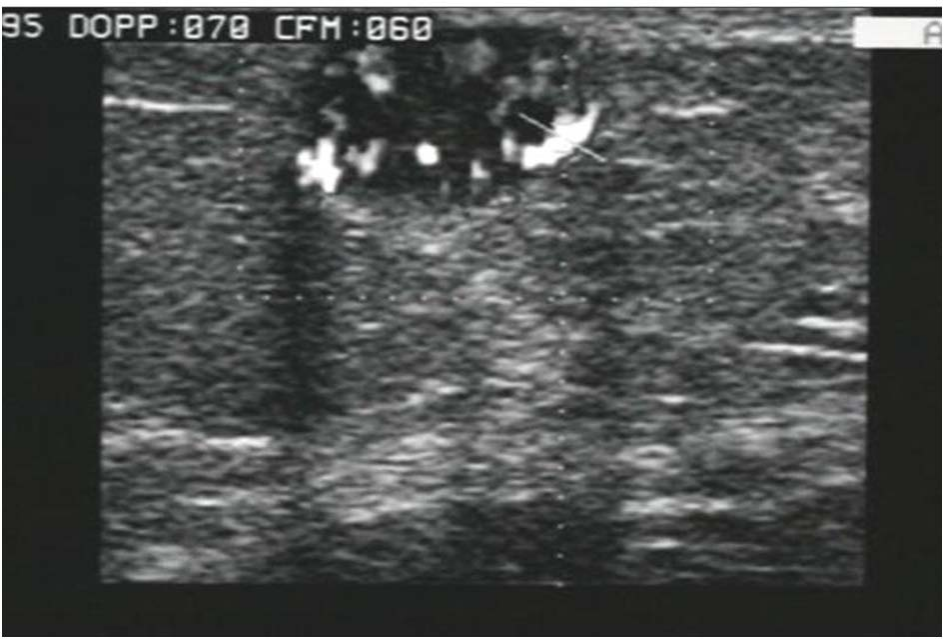
**Pattern type 5: Pseudo-neoplastic**, solid nodulation, hypoechogenic, not homogeneous, with irregular anterior contours, with signal with Colour and Power-Doppler.

**Figure 6:**
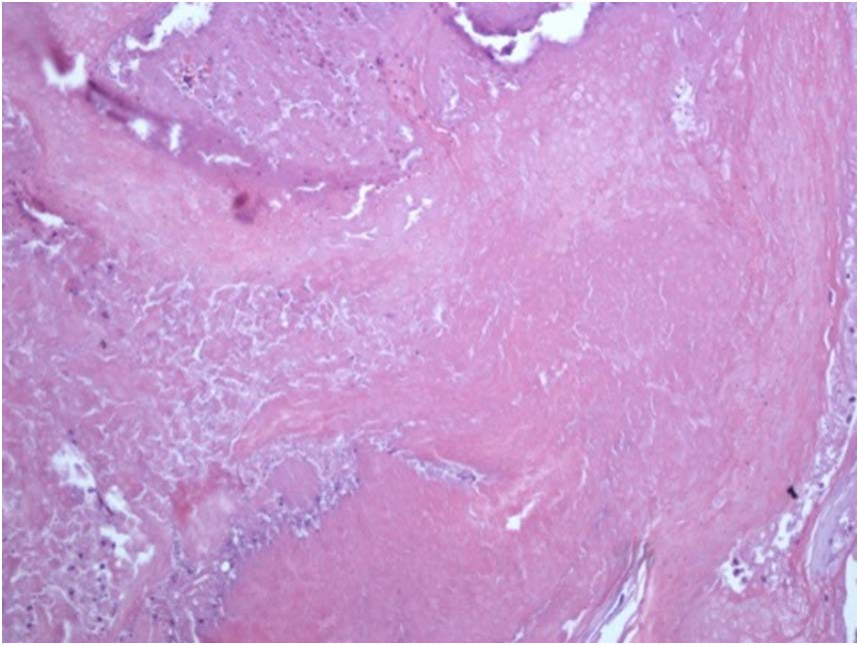
**Shadow cell and thricholemmal keratinization details, interspersed inflammatory cells (E-E 20×)**.

In 3 uncertain diagnosed cases, a complex ultrasound lesion (mixed pattern) was found, with mixed fluid and solid areas, scattered microcalcifications, and some signals to the colour Doppler (Fig. 3). The 7 misdiagnosed cases included 3 mixed pattern lesion, 2 cystic-like (Fig. 4) and 2 solid, vascularised nodules with irregular contours (Fig. 5) (Table. 2-US findings of pilomatricomas). Figure 6 shows a detail of the histological pattern of these tumor.

**Table 2:** US findings of pilomatricomas

Type	US features	No. of lesions
Type 1	*Fully calcified*	10
Type 2	*Partially calcified*	12
Type 3	*Complex lesion*	6
Type 4	*Pseudocystic lesion*	2
Type 5	*Pseudotumoural*	2

Finally, 2 lesions, with pseudo-neoplastic features, were also studied with a second generation contrast medium (SonoVue, Bracco, Milan, Italy), injected via a bolus in the antecubital vein, and showed moderate enhancement of the lesion and the presence of rather irregular internal vessels. The most experienced radiologist (30 years of general ultrasound and 11 of dermatological ultrasound), assessed a correct diagnosis in 11/15 cases (74%), misdiagnosed in 2/15 cases (13%) and provided a non conclusive response in the remaining 2/15 cases (13%). There were no significant differences (p = ns) among experienced and less experienced radiologists in diagnosing PM.

Due to the small size of the lesions and to the need for immediate surgical treatment, none of our patients were studied by CT scan or MRI.

Only 1 case of multiple PM (5 lesions in the same patient) was found, and the genetic examination excluded the coexistence of myotonic dystrophy.

## Discussion

PM is an uncommon cutaneous tumour affecting young adults, especially women. It originates from the matrix cells of the hair follicle.

Despite their benign behaviour, very malignant forms have been reported in literature. So far, most of the studies have revealed the difficulties encountered in diagnosing PM clinically. Imaging techniques such as X-ray, CT scan, MRI, and FNAB have failed to differentiate PM from other pathologies. Ultrasounds have only been of significant use in detecting bigger lesions, and most of the authors evaluated images obtained from low-frequency ultrasound (7.5-10 MHz). Since the probe resolution power is a direct proportional function of the frequency used, a very high frequency must be employed to characterize small lesions such as PM. In particular, the following data, provided from the Esaote Research Centre of Genoa, concerning the real experimental resolution power of their manufactured ultrasonographic probes: 7.5 MHz linear probe: axial resolution 0.2, lateral resolution 0.25; 10 MHz linear probe: axial resolution 0.154, lateral 0.187; 13 MHz linear probe: axial resolution 0.188, lateral resolution 0.144; 18 MHz linear probe: axial resolution 0.085, lateral resolution 0.104; 20 MHz annular array: axial resolution 0.077, lateral resolution 0.094.

In our study, we have reviewed 32 series of images obtained from high-frequency ultrasound units and have found 5 sonographic patterns to differentiate PM from other subcutaneous tumours. In particular, Type 1 and 2 of our classification correspond to the two typical hypoechoic solid nodules, fully calcified and partially calcified respectively, already described in literature. These lesions normally present a hypoechoic peripheral rim in a significant number of cases, and rarely, vascular signals with colour Doppler.

In our series, 22 lesions exhibited the solid and calcified patterns of type 1 (10 cases) and 2 (12 cases), and diagnosis was confirmed at histopathology.

Eight cases (25%) of our series showed internal fluid areas with a thick-wall: 6 complex lesions (type 3) and 2 pseudo-cystic (type 4). Type 4 fluid areas were larger than type 3 and showed a good transmission of the ultrasound wave, without enhancement of the posterior wall. Histologically, the pseudo-cystic lesions showed huge groups of ghost cells, without stroma, clearly correlated to the sonographic features.

Lim et al. [20] described 2 cases out of 17 with little endotumoural liquid-like areas, which the author, and, more recently, Choo et al. [30], considered to be related to degenerative phenomena. We are the first to report the occurrence of real ultrasonographic cystic areas in PM.

As pointed out by some dermatopathologists [31], the tumour originates from a cystic formation of the follicle matrix, with more or less thick walls, depending on the neoplasia evolvement, and with consequential formation of an internal mass of shadow cells, with low vascularisation and almost absent stroma. Generally, calcifications and signs of inflammation appear belatedly.

The homogeneity of pseudo-cystic fluid areas, the lack of internal interfaces and of fibrous support structures, the absence of internal signs with colour Doppler, but without enhancement of the posterior wall, might address the operator to an erroneous diagnosis. The resemblance of sonographic features to so-called sebaceous cysts (epidermal or trichilemmal cysts), might result from the very high frequency probes that we first used in this particular type of dermopathology. Two cases, with a tumour-like pattern (type 5), were indistinguishable from an aggressive neoplasia of the superficial structures; in both patients, the lesions were significantly old and, histologically, displayed chronic flogistic phenomena and fibrosis.

## Conclusion

Based on the above, some remarks can be drawn:

1 -Using very high frequency probes, we have identified five different ultrasound patterns of PM. Pattern type 3, 4 and 5 have never been described before, not even in the recent paper of Choo et al. [30].

This finding constitutes a new important contribution that deserves to be promptly shared with other specialists working in the field:

• type 1, 31.5% of cases; nodule fully calcified, semi-superficial with minimal solid hypoechoic peripheral ring, with an average size of 11 mm (Fig. 1);

• type 2, 37.5% of cases; nodule partially calcified, with internal calcareous formations, of variable size (average diameter of 10 mm), with a solid hypoechoic peripheral component, avascular (Fig. 2);

• type 3, complex formation; 19% of cases (Fig. 3);

• type 4, 6% of cases; pseudocystic formation, without enhancement of the posterior wall, with semi thick walls (Fig. 4)

• type 5, 6% of cases; pseudo-neoplastic nodules; the inflammatory phenomena seemed to justify the pattern (Fig. 5).

2-As described in literature, the diagnostic accuracy of an experienced operator is very high for "classic" forms, but it is lower for the three new patterns.

3-There were no differences in the evaluation of the features of the images among less experienced and expert radiologists. This evidence could be explained by the relatively high incidence of lesions with non-classical patterns encountered in our series.

4-We used higher resolution apparatus, that certainly permitted good performances in the diagnosis of the "classic" forms, but showed better results in discriminating the peculiar characteristics of pattern 3, 4 and 5. However, more cases would be needed to evaluate the real incidence of those new patterns.

5-Although our results showed only 69% of correct diagnosis compared to 96% (50/52) of Whittle et al. [28] and 82% of Lim et al. [20] (17/18), we reached 100% when considering only the "classic" forms (pattern 1 and 2), which are really easily diagnosable with ultrasounds.

6-In agreement with Choo et al. [30], and for the few cases we studied, the colour-power Doppler and the second generation contrast media did not seem to give significant diagnostic advantages. In conclusion, we believe, that the knowledge of these three new patterns, not previously described, could help in the clinical diagnosis of pilomatricoma, and, consequently, in the diagnostic and therapeutic management of this type of neoplasia.

## Competing interests

The authors declare that they have no competing interests.

## Authors' contributions

FE and AD carried out the research, participated in the sequence alignment and drafted the manuscript text. CP and AA assessed the pathological diagnosis. ADC contributed with his professional experience to the revision of the manuscript. FMS conceived the study, participated in its design, carried out the research and coordinated the study. All authors read and approved the final manuscript.
